# A Chinese classical prescription Maimendong decoction in treatment of pulmonary fibrosis: an overview

**DOI:** 10.3389/fphar.2024.1329743

**Published:** 2024-05-09

**Authors:** Qiurong Lao, Xianbin Wang, Guangqing Zhu, Haochen Yuan, Ting Ma, Ning Wang

**Affiliations:** ^1^ College of Traditional Chinese Medicine, Shandong University of Traditional Chinese Medicine, Jinan, China; ^2^ College of Rehabilitation Medical, Shandong University of Traditional Chinese Medicine, Jinan, China; ^3^ Research Department of Shandong University of Traditional Chinese Medicine, Jinan, China

**Keywords:** pulmonary fibrosis, Maimendong decoction, classical prescription of traditional Chinese medicine, mechanism, pharmacological effects

## Abstract

Pulmonary fibrosis (PF) is a chronic and progressive disease characterized by fibrosis and interstitial pneumonia. It has similar clinical symptoms to “Fei Bi” and “Fei Wei” as described in the traditional Chinese medicine (TCM) classic *Jingui Yaolue* written by Zhang Zhongjing in the Han Dynasty. This study explored the potential of Maimendong Decoction (MMDD). MMDD consists of Ophiopogon japonicus (L.f) (*ophiopogonis*), Pinellia ternata (Thunb.) Breit. (*pinellia*), Panax ginseng C. A. Mey. (*ginseng*), Glycyrrhiza uralensis Fisch. (*glycyrrhiza*), Zizi phus jujuba Mill. (*jujuba*), and Oryza sativa L. (oryza sativa), with the function of nourishing the lung and stomach, and reducing the effect of reverse qi. It has been used clinically for over two thousand years to treat conditions like “Fei Bi” and “Fei Wei”. Previous research suggests that MMDD and its individual herbal extracts have anti-fibrotic effects. The main focus of MMDD in treating PF is to reduce inflammatory cytokines, inhibit pro-fibrotic factors and oxidative stress, promote differentiation and homing of bone marrow mesenchymal stem cells, and enhance cell autophagy activity. This review summarized the clinical applications, mechanisms, and pharmacological effects of MMDD in treating PF based on existing clinical applications and experimental research. It also discussed current issues and prospects, aiming to provide a reference for further research on the mechanism of PF, drug development, and clinical trials.

## 1 Introduction

Pulmonary fibrosis (PF) is a chronic and irreversible lung condition classified under interstitial lung diseases. It involves the scarring and thickening of lung tissue. Various triggers, including environmental factors, occupational exposure, medications, autoimmune diseases, and genetic factors, can cause PF. Common symptoms experienced by PF patients include shortness of breath, persistent dry cough, wheezing, sputum production, chest tightness, and chest pain (Mathai and Schwartz, 2019; Liu et al., 2022). In recent years, the incidence of PF is more than 100,000 people each year, and it has been rising sharply (Pan et al., 2020). Among them, idiopathic PF, in particular, has garnered public attention due to its increasing prevalence and incidence worldwide (Wijsenbeek, 2020; Maher et al., 2021). The annual incidence of idiopathic PF reaches as high as 17.4 people per 100,000 (Zhao et al., 2020), with an average survival after diagnosis of only 2.8 years. The mortality rate exceeds that of most tumors (Viale, 2020), and complications are common. PF patients also face a significantly higher risk of lung cancer compared to the general population, further impacting their quality of life and burdening society (Tzouvelekis et al., 2020). Consequently, prevention and treatment have become urgent priorities within the medical community. Currently, Western medicine primarily employs glucocorticoids (Wiertz et al., 2018), nintedanib, pirfenidone, and other drugs to slow down disease progression (Richeldi et al., 2020). However, these treatments cannot offer a complete cure, often carry significant side effects, are expensive, and lack widespread acceptance among the general public (Cottin et al., 2023).

**FIGURE 1:**
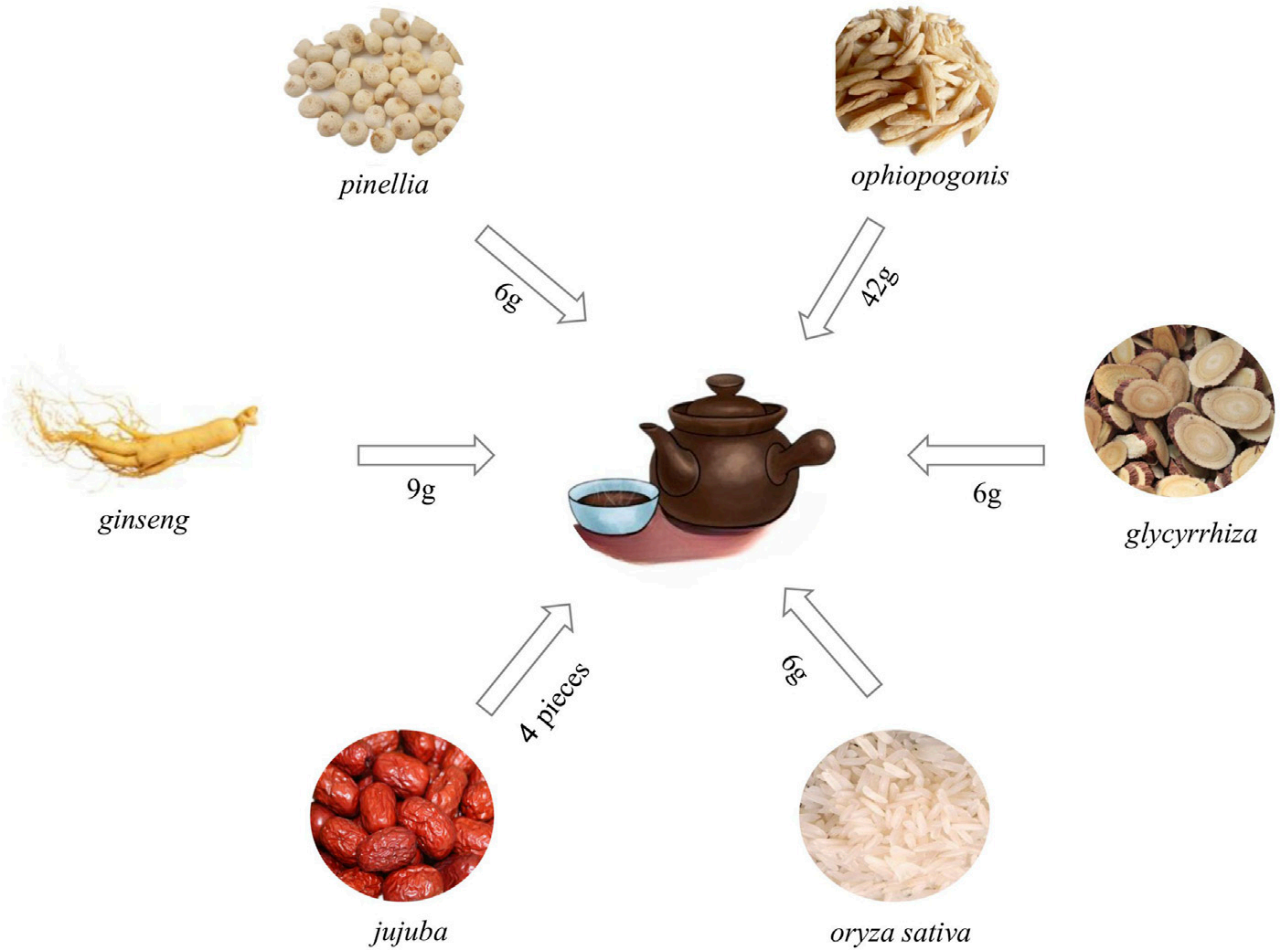
Composition and dosage of Maimendong Dection (Maimendong Dection composed of *ophiopogonis*, *pinellia*, *ginseng*, *glycyrrhiza*, *jujuba* and *oryza sativa*).

In recent years, with the deepening of Chinese medicine research, the study found that Chinese medicine in the treatment of PF in a significant effect, and minimizing side effects (Guo et al., 2019). TCM categorizes PF as a manifestation of “Fei Bi” and “Fei Wei,” as documented in the *Jingui Yaolue*: “Cunkou pulse rapid, who coughs and has turbid saliva in the mouth. Teacher said: for the disease of Atrophy of the lung lobes.” According to this perspective, the lungs are closely connected to the spleen and kidneys. Exogenous etiological factors, seven emotions, sexual overindulgence and mistreatment contribute to the development of PF. The pathogenesis is characterized by deficiencies in healthy qi, yin deficiency leading to hyperactivity of fire, lung yin deficiency pattern, phlegm and blood stasis obstructing the lung. Consequently, the syndrome is categorized as originating from deficiency with superficial excess. Clinical treatment can tonify lung qi, resolve phlegm, promote blood circulation, and remove blood stasis (Zang et al., 2017; Cheng et al., 2020; Li et al., 2022). Maimendong Decoction (MMDD), a classic prescription from the *Jingui Yaolue*, consists of Ophiopogon japonicus (L.f) (*ophiopogonis*), Pinellia ternata (Thunb.) Breit. (*pinellia*), Panax ginseng C. A. Mey. (*ginseng*), Glycyrrhiza uralensis Fisch. (*glycyrrhiza*), Oryza sativa L. (oryza sativa), and Zizi phus jujuba Mill. (*jujuba*) (Figure 1). It has the effect to nourish and supplement lung and stomach, and reduce the effect of reverse qi. It has long been used to treat cough, lung cancer, PF, and other respiratory diseases (Yulong and Chunshui, 2017). Due to the influence of various factors like environment, genetics, and lifestyle, the prevalence of PF continues to rise annually. Consequently, there is an urgent need to identify effective therapeutic solutions (Bhavana et al., 2021). While MMDD has been widely employed in PF treatment, its precise mechanism remains unclear. Therefore, this systematic review was aimed to provide a comprehensive analysis of clinical application, mechanism, pharmacological action, and effective material basis of MMDD in treating PF, enabling a deeper understanding of its pharmacological mechanism and prescription characteristics. This review will serve as a reference for the clinical development of new drug treatments for PF.

## 2 Methodology

Keywords, including Maimendong, Maimendong Decoction, Pulmonary Fibrosis, Idiopathic Pulmonary Fibrosis, Idiopathic Diffuse Interstitial Pulmonary Fibrosis, Fibrosis, lung, were used to search related literature and data through digital resources and paper-based materials. The earliest available documents were published in 1968, while the latest was published in April 2024. These documents discussed the clinical randomized controlled trials of MMDD in the treatment of PF and the mechanism of MMDD in the treatment of PF. Most of the literature research was conducted through the following eight online scientific databases: PubMed, Web of Science, Google Scholar, SciFinder, CNKI, Baidu Scholar, China Science and Technology Journal Database, Wan Fang Data Knowledge Service Platform. The review also included results from Ph.D. theses, M. Sc. Dissertations.

## 3 Clinical applications of MMDD in the treatment of PF

Modern studies have demonstrated that modified MMDD or its combination with Western medicine can effectively treat PF with fewer side effects. It has been considered as an effective treatment for PF. For instance, Bai et al. (2019) conducted a study with 84 PF patients divided into control and treatment groups. The control group received pirfenidone capsules, while the treatment group received pirfenidone capsules along with MMDD. The researchers found that both groups experienced reduced TCM syndrome scores after treatment. Additionally, the forced vital capacity (FVC), carbon monoxide diffusion volume (DLCO), and forced expiratory volume occupancy lung capacity ratios increased, while levels of hyaluronic acid (HA), laminin (LN), and type Ⅲ precollagen (PCⅢ) decreased. Scores on the George Respiratory Questionnaire (SGRQ) increased in all aspects, indicating significant improvements in the treatment group compared to the control group. These findings suggest that the therapeutic effects of combining pirfenidone with MMDD were superior to pirfenidone capsules alone. Wong and Qu (2008) conducted a clinical observation involving 48 PF patients to compare the efficacy of MMDD and glucocorticoids. Pulmonary function and arterial oxygen partial pressure data showed better indicators for the MMDD group than the glucocorticoid group. Furthermore, the total effective treatment rate was 81.25% in the MMDD group, while it was 56.25% in the glucocorticoid group, indicating that MMDD was more effective than glucocorticoids in treating PF. Yu (2010) randomly assigned 60 PF patients to a control group and a treatment group. The control group received prednisone, while the treatment group received modified MMDD (consisting of *ophiopogonis, pinellia, ginseng, Baked glycyrrhiza, oryza sativa, jujuba, Astragali radix, Schisandrae Chinensis Fructus, Cornus Officinalis, Gecko, Chuanxiong Rhizoma*, and *Pheretima*) orally for 3 months in addition to prednisone. The study measured the FVC and DLCO of the patients before and after treatment, and scored improvements in symptoms such as cough, sputum volume, sleep, fatigue, and number of breaths per minute. The nimodipine score showed that the MMDD plus prednisone group had better scores than the prednisone group, indicating that the combination of MMDD and Western medicine was more effective than Western medicine alone. Gan et al. (2020) intervened with modified MMDD and pirfenidone capsules in PF patients, while the control group received placebo and pirfenidone capsules. The study evaluated the efficacy and safety of modified MMDD and pirfenidone capsules by observing average changes in lung capacity, the number of acute exacerbations, baseline changes, liver and kidney function, and other laboratory tests through a double-blind, randomized trial. The researchers initially determined that MMDD and pirfenidone capsules could improve lung function, reduce the duration of acute exacerbation, and enhance the quality of life in patients. Liu (2017) studied the qi and yin deficiency syndrome in PF patients. The control group received oxygen inhalation, anti-inflammatory treatment with prednisone, and pirfenidone, while the treatment group received MMDD in addition to the control group’s treatment. After 2 months, the treatment group showed better total scores for symptoms and signs (cough, asthma, expectoration, spontaneous sweating, hot flashes, night sweats, five upset heat, and velcro rales) compared to the control group. The treatment group also exhibited improved lung function levels and significantly higher scores in each dimension of the short form 36 health survey questionnaire (SF-36) scale, indicating that MMDD had a therapeutic effect on qi and yin deficiency syndrome in PF patients. The effects were superior to the conventional Western medicine treatment alone. These clinical studies provide evidence that MMDD has significant therapeutic effects on PF (Table 1). Moreover, MMDD, either used alone or in combination with Western medicine, demonstrates better efficacy and fewer side effects compared to Western medicine alone. Therefore, MMDD holds promise as a future treatment modality for PF.

**TABLE 1:** Clinical application of MMDD in the treatment of PF.

	Group	Medication (dosage of drug)	Number of people	Age (average age)	The medication time	Inclusion time	Inclusion time	Data sources	Treatment effect	References
1	control group	The control group was treated with pirfenidone capsules	42	37–76 (56.48 ± 6.71)	200 mg per time, 3 times a day; 3 consecutive months	June 2017-July 2018	7 months-5years (2.96 ± 0.84years)	Department of Respiratory, Affiliated Hospital of Traditional Chinese Medicine, Xinjiang Medical University	the measured values of FVC, FEV1/FVC and Dlco were increased in both groups (*p* < 0.05), and the increase was more significant in the treatment group (*p* < 0.05); The HA, LN and PCⅢ decreased in both groups (*p* < 0.05), and the decrease was more significant in the treatment group (*p* < 0.05). The scores of SGRQ in all aspects were increased in both groups (*p* < 0.05), and the increase was more significant in the treatment group (*p* < 0.05)	Bai et al. (2019)
	treatment group	The treatment group added MMDD (Maidong35g, oryza sativa 20 g, Astragalus20 g, Ginseng15 g, Schisandra15g, Dogwood15g, Clam15g, Chuanxiong15g, glycyrrhiza 15 g, Dilong10 g, Banxia5 g, Jujube3) on the basis of pirfenidone capsules	42	38–74 (56.29 ± 6.53)	One dose per day, 300 mL decocted per dose,3 times a day for 3 months		8 months-5 years (2.97 ± 0.82 years)			
2	control group	oral prednisone tablets	16	39–79 (51.8)	oral prednisone tablets 30–60 mg/d for 1 month	September 2001-May 2007	3 months	Henan Nanyang Medical College	The control group had an effective rate of 56.25%, the effective rate of the treatment group was 81.25%. After treatment, the arterial partial pressure of oxygen increased in both groups, and lung function (VC,DLco)improved, but the treatment effect of the treatment group was better than that of the treatment group	Wong and Qu (2008)
	Treatment group	modified MMDD (MaiDong60 g, ginger banxia20 g, raw sundried ginseng15g, glycyrrhiza 12g, glutinous rice30 g, jujube4{qi deficiency plus astragalus30 g, white art30 g; Add 12 g of dried ginger and 1 clam; Add 12 g of Panax ginseng for heavy stasis, 9 g of cannon armor powder (punching); phlegm fuller plus chuanbei16g, gallbladder star9g; Those with fever add 20 g of skullcap and 30 g of houttuynia corido	32	38–75 (49.8)	One dose per day, decoction 750 mL per dose, 250 mL each time, 3 times a day, for 1 month					
3	Control group	prednisone	30	39–73 (61.5 ± 10.9)	The initial dose is 0.5 mg/kg for 4 weeks, then 0.24 mg/kg daily for 8 weeks, and then reduced to 0.125 mg/kg per day	June 2008-February 2010	6 months-4.2 years (2.7 ± 1.5 years)	Affiliated Hospital of Liaoning University of Traditional Chinese Medicine	The two groups had significant improvement (*p* < 0.05) after treatment compared with before treatment, and the treatment group was significantly better than the control group (*p* < 0.05), and the lung function (FVC,DLco)after treatment (FVC,DLco) was significantly improved (*p* < 0.05) between the treatment group and the control group compared with the pre-treatment group, and there was no significant difference between the two groups after treatment (*p* > 0.05). It showed that both groups could improve lung function and the efficacy was comparable	Yu (2010)
	treatment group	The treatment group took modified MMDD (Mai Dong 35g, Qing Banxia 5 g, Ginseng 15g, glycyrrhiza 15g, oryza sativa 20 g, Jujube 7 Jujubes, Astragalus 20 g, Schisandra 15g, Mountain Mussel 15g, Clam 15g, Chuanxiong 15 g, Dilong 10 g)	30	38–75 (63.8 ± 11.5)	Decoction 300 mL per dose,3 times a day for 3 months		4.8 months - 4.1 years (2.2 ± 1.2 years)			
4	control group	The control group was given placebo granules plus pirfenidone capsules	30	18–75	Placebo granules 1 sachet, and 3 times a day, each bag of granules dissolved in 100 mL of boiled water for 24weeks), pirfenidone capsules (200 mg each time for the first week, 3 times a day for 1 week; For the second week, 400 mg three times daily for 4 weeks.600 mg 3 times daily for 18 weeks for week 6)	September 2020-September 2021		Affiliated Hospital of Chengdu University of Chinese Medicine	The addition or subtraction of Maimendon tong plus pirfenidone capsules can improves lung function, reduces the duration of exacerbations, and improves quality of life in patients with pulmonary fibrosis	Gan et al. (2020)
	treatment group	The treatment group was given MMDD granules and add pirfenidone capsules (Maidong10 g, Banxia15 g, Ginseng5 g, Knotweed10 g, Chuanxiong10 g, Danshen15 g glycyrrhiza 5 g)	30	18–75						
5	controlgroup	The control group was given oxygen, prednisone anti-inflammatory, and pirifenidone	20	54.49 ± 13.386	Pirfenidone 200 mg/time in the first week, 3 times/day, prednisone 0.5 mg/kg daily for 4 weeks; Then 0.25 mg/kg daily orally for 8 weeks; Then reduce the dose to 0.125 mg/kg daily or 0.25 mg/kg orally once every other day, as appropriate	December 2015-November 2016	4.25 ± 0.813	The First Affiliated Hospital of Traditional Chinese Medicine of Hunan Province	After 2 months, the total points of symptoms and signs (cough, wheezing, sputum production, self-sweating, hot flashes and night sweats, five upset fevers, velcro rales) in the treatment group were better than those in the control group.The treatment group improves the level of lung function; In improving the scores of all dimensions of the SF-36 scale, the treatment group was significantly better than the control group	Liu (2017)
	treatment group	The treatment group was given modified MMDD (Maidong 15 g, Codonopsis 15 g, Faban Xia 10 g, Nansha ginseng 15 g, Baishu 10 g, Pori 15 g, Yuzhu 10 g, smallpox powder 10 g, Astragalus 15g, Knotweed 10 g, Chuanxiong 10 g, Danshen 15 g, psoralen 10 g, glycyrrhiza 5 g)	20	54.68 ± 13.516	one dose per day, twice daily, divided in the morning and evening		4.50 ± 0.892			

However, from the existing research, it is found that MMDD is mainly used in China, Japan, South Korea and other countries. It has not been found whether MMDD has been approved for use in Western countries. Therefore, in the future, the benefits of traditional Chinese medicine can be popularized and promoted, so that traditional Chinese medicine can play a role in the health of all mankind.

## 4 Mechanism of MMDD in the treatment of PF

### 4.1 Reduction of inflammatory cytokine levels by MMDD

PF is characterized by inflammatory injury, with inflammation playing a crucial role throughout its progression. Interleukins (ILs) and other inflammatory mediators have been confirmed to be involved in PF, reflecting its degree of inflammation, prognosis, and severity assessment (She et al., 2021). Tumor necrosis factor-α (TNF-α) activity and the presence of inflammatory cells are pro-inflammatory factors that stimulate chemotaxis of inflammatory cells and promote local inflammatory responses (Tang et al., 2011). Therefore, reducing levels of inflammatory cytokines is an effective strategy for treating PF. IL-10 exerts anti-inflammatory effects by inhibiting TNF-α production at the transcriptional level (Islam et al., 2022). Studies have shown that MMDD can reduce alveolar inflammation and PF in rats, potentially through the decrease in IL-10 expression and inhibition of lung TNF-α, transforming growth factor-β1 (TGF-β1), platelet-derived factor (PDGF), and connective tissue growth factor (CTGF) (Du, 2015). Huang et al. (2020) found demonstrated that the combination of MMDD and *Shenling Baizhu Powder* can significantly reduce the protein content and gene expression of high mobility group protein 1 (HMGB1), IL-6, PDGF, and K-Ras in lung tissue. They also found a positive correlation between HMGB1 and PDGF, suggesting that TCM treatment for silicosis fibrosis involves the HMGB1/PDGF/Ras signaling pathway. Li et al. (2009) observed that MMDD can significantly reduce alveolitis caused by pingyangmycin in rats, increase the expression of IL-10 in lung tissue, and inhibit the overexpression of TNF-α. He et al. (2024) found that MMDD could improve the body weight and reduce the inflammatory response of PF model mice, and the possible mechanism was that MMDD inhibited M2 macrophage polarization, released profibrotic factors, and inhibited PF in rats.

### 4.2 Inhibition of profibrotic factor expression by MMDD

TGF-β is a cytokine that contributes to the development of PF and plays a role throughout its progression (Ghatak et al., 2017). It promotes the activation and differentiation of inflammatory cytokines in the early stage and the division and proliferation of fibroblasts in the later stage, ultimately leading to collagen synthesis and ECM (ECM) deposition, resulting in thickening of the alveolar wall and PF(Stewart et al., 2018). Therefore, inhibiting the expression of TGF-β and its related factors can slow down PF progression. Yuan et al. (2023) observed improved lung function in patients with silicosis-induced fibrosis following intervention with miR-200 bagomir. This intervention alleviated protein concentration, total white blood cell count, and TGF-β1 content in bronchoalveolar lavage fluid. Liu et al. (2022) investigated the effects of modified MMDD on mice with bleomycin-induced PF. They found that the modified MMDD group exhibited reduced mortality and lung coefficient compared to the model group. Pathological changes such as inflammatory cell infiltration, severe alveolar structure destruction, and collagen deposition in lung tissue were also reduced. Moreover, the protein and mRNA expression of TGF-β1, type I collagen (COL1A), smooth muscle actin (α-SMA), phosphorylated (p)- Phosphoinositide 3-kinase (PI3K), p-protein kinase B (AKT), and mTOR were decreased, indicating that modified MMDD could effectively improve bleomycin-induced PF. The mechanism may involve the regulation of the PI3K/AKT/mTOR signaling pathway, inhibition of epithelial mesenchymal transition, and reduction of ECM deposition. Zhang et al. (2017) administered MMDD to rats with PF and found that it significantly inhibited the expression of Smad3 and TGF-β1 protein in the lung tissue of the model group while increased the expression of Smad7 protein. These results suggest that MMDD may improve the pathological state of PF in rats by regulating the expression of TGF-β1, Smad3, and Smad7 proteins. Liu et al. (2018) proposed that MMDD alleviated alveolar inflammation and fibrosis by inhibiting the protein expression of TGF-β1, matrix metalloproteinase (MMP-9), and matrix metalloproteinase tissue inhibitor 1 (TIMP-1) in lung tissue, thereby regulating abnormal ECM metabolism. Wang (2017) suggested that reducing the expression of TGF-β1 and ColI may alleviate lung tissue damage, alveolar inflammation, and collagen fiber formation induced by bleomycin, subsequently reducing the development of PF. Xu et al. (2024) found that mice in the model group had elevated lung coefficients, a large number of inflammatory cell infiltration and collagen fiber deposition in lung tissues, and elevated lung fiber scores, and after administration of MMDD, mice had reduced lung coefficients, decreased inflammatory cell infiltration and collagen fiber deposition in lung tissues, and reduced lung fiber scores, and Collagen I, TGF-β1, p-STAT3, PD-1, PD-L1, and IL-17A decreased under expression, suggesting that maitake soup retarded the process of PF by reducing ECM deposition, and the mechanism may be related to with the inhibition of STAT3/PD-1/PD-L1 immunoregulatory signaling pathway.

### 4.3 Inhibition of oxidative stress damage by MMDD

Exposure of lung tissue to high oxygen concentrations can lead to oxidative stress damage. When the body’s production of reactive oxygen species (ROS) is excessive or antioxidant capacity is insufficient, ROS accumulation induces oxidative stress, resulting in lung tissue damage and remodeling. Hence, inhibiting oxidative stress damage is crucial for preventing and alleviating PF(Fois et al., 2018). Chen (2020) induced two mouse models of PF using trans-resveratrol (TR) and bleomycin, respectively, and treated both groups with MMDD. The results showed significant differences in the treatment effects between the two models. The TR model mice exhibited higher weight and lung coefficient, while the bleomycin model showed better pathological changes. The activity levels of superoxide dismutase (SOD) and glutathione peroxidase (GSH-PX) increased in the TR model but decreased in the bleomycin model. Additionally, the decrease in malondialdehyde (MDA) in lung tissue was significantly higher in the TR model. The levels of IL-13 and IL-4 decreased to varying degrees, with IL-13 decreasing more in the bleomycin model and IL-4 decreasing more in the TR model. TNF-α content increased in the TR model but showed the opposite trend in the bleomycin model, demonstrating that MMDD had a therapeutic effect on both PF models, potentially through oxidative stress regulation. Zhang et al. (2012) explored the intervention mechanism during the formation stage of PF and found that MMDD treatment resulted in higher SOD activity and lower MDA content in serum and bronchoalveolar lavage fluid of rats. This indicated that MMDD had an inhibitory effect on the decrease of SOD activity and increase of MDA content in lung tissue and serum of model rats. Liu et al. (2019) induced fibrosis in human embryonic lung epithelial cells (MRC5 cells) using TGFβ1 and then intervened with MMDD-containing serum and the peroxisome proliferator-activated receptor (PPARγ) antagonist GW9662. They observed that MMDD-containing serum reduced the increase of soluble collagen induced by TGFβ1 stimulation, increased PPARγ activity, and inhibited intracellular ROS damage. Conversely, the PPARγ antagonist GW9662 inhibited PPARγ activity induced by MMDD-containing serum. Animal experiments showed increased PPARγ expression in lung tissue and decreased soluble collagen content, suggesting that the mechanism of MMDD in treating PF may be related to the synthesis of fibrogenic factors and collagen (Liu et al., 2019). It was found that excessive absorption of molybdenum in humans may cause PF, and its mechanism of action is related to inhibition of the SLC 7A 11/GSH/GPX 4 axis leading to iron death, increased CAV-1 expression, and activation of the Wnt/β-linker pathway (Lipner et al., 2022).

### 4.4 Promotion of the differentiation and homing of bone marrow mesenchymal stem cells (BM-MSCs) by MMDD

Bone marrow has the ability to generate fibroblasts that promote PF, but it also produces mesenchymal stem cel ls (MSCs) that possess anti-fibrotic properties. Derived from bone marrow, BM-MSCs are pluripotent stem cells with self-proliferation and multidirectional differentiation potential (Friedenstein et al., 1968). These cells can play a role in preventing fibrosis through their homing and differentiation abilities (Zhao et al., 2019). Lei (2018) observed lung inflammatory responses and collagen fiber expression in rat lung tissues, finding that MMDD effectively treated PF by reducing lung inflammatory infiltration and collagen fiber deposition. This effect was associated with the mobilization and differentiation of BMSCs, as well as the expression of aquaporin-5 (AQP-5), Surfactant protein C (SP-C), and their mRNA. Liu et al. (2018) used a similar method and discovered that MMDD promotes the expression of CD44 and CD90 positive cells in peripheral blood through BMSC cell mobilization, which might be one of the mechanisms of action. Liu (2019) found that MMDD or MMDD-containing serum medium combined with serum-free small airway epithelial cell culture medium can induce BMSCs to differentiate into type Ⅱ alveolar epithelial cells (AEC2s) by activating the Wnt/β-catenin signaling pathway. This indicates that the mechanism behind MMDD’s treatment of PF is related to the Wnt/β-catenin signaling pathway. He (2019) investigated whether MMDD-treated BMSCs could improve PF tissue repair through the Stromal cell-derived factor-1 (SDF-1)/C-X-C chemokine receptor type 4 (CXCR4) axis. The results showed that the MMDD group significantly increased the mRNA and protein expression of SDF-1, while the addition of AMD3100 to block the combination of SDF-1 and CXCR4 inhibited the improvement effect. Compared to the BMSCs group, the MMDD-BMSCs group exhibited reduced PF area, decreased inflammatory cell infiltration, and increased homing of BMSCs. This suggests that MMDD enhances the directional homing of BMSCs to injured lung tissue, promotes tissue regeneration, and reduces fibrosis by increasing the number of homing BMSCs. These effects may be associated with the SDF-1/CXCR4 axis. Liu et al. (2019) observed the effect of MMDD-containing serum on the differentiation of BMSCs into alveolar epithelial cells in rats. The results indicated that the protein expression of AQP-5 and green fluorescent protein (GFP) -BMSC cell SP-C was significantly higher in the MMDD group compared to the model group, suggesting that MMDD can promote the differentiation of GFP-BMSCs into alveolar epithelial cells, which may be one of the mechanisms behind its treatment of PF. Shen (Shen et al., 2020) found that MMDD could alleviate the pathological process of PF to some extent, improve lung function in rats with PF, reduce ECM deposition, and inhibit the transformation of fibroblasts into myofibroblasts. They speculate that MMDD treated PF mainly by reducing the inflammatory reaction in lung tissue of fibrotic rats, thereby reducing ECM collagen content, and increasing the secretion of active SP-C by AECⅡ to the alveolar surface.

### 4.5 Enhancement of autophagy activity by MMDD

Autophagy is an adaptive cellular process that helps protect against various forms of stress, such as nutrient deficiency, growth factor deprivation, infection, and hypoxia, thereby preventing cell damage (Hu et al., 2022). Studies have shown that autophagy can delay the progression of PF(Shi et al., 2021). Zhao et al. (Zhao et al., 2021) treated mice with interstitial pneumonia using different concentrations of MMDD and found that compared to the model group, the lung tissue wet/dry weight ratio (W/D) decreased and the alveolar fluid clearance rate increased in each dose group of MMDD. The high and middle dose groups exhibited clearer alveolar structures, reduced collagen protein levels, and improved PF, while the low dose group showed evident PF and collagen deposition. The expression of α-SMA, p-PI3K, p-AKT, p62, and p-mTOR proteins in type Ⅱ alveolar epithelial cells decreased in each dose group, while the expression of light chain 3 (LC3)-Ⅱ protein increased. These findings suggest that MMDD has a therapeutic effect on interstitial pneumonia mice, with the therapeutic effect being closely related to the drug dosage. The mechanism behind this effect involves the PI3K/AKT/mTOR pathway and the enhancement of autophagy activity in lung tissue cells. Based on network pharmacology and experimental verification, Wang et al. (2022) found that MMDD treatment of PF had lower binding energy with β-sitosterol, stigmasterol, kaempferol, protopine, betulinic acid, quercetin compounds and targets AKT1, GAPDH, STAT3, MAPK3, indicating that these compounds have potential inhibition of pulmonary fibrosis activity; *in vitro* experiments showed that MMDD could promote the expression of AKT1, p-AKT protein and downregulate the expression of VFGF protein.

**FIGURE 2:**
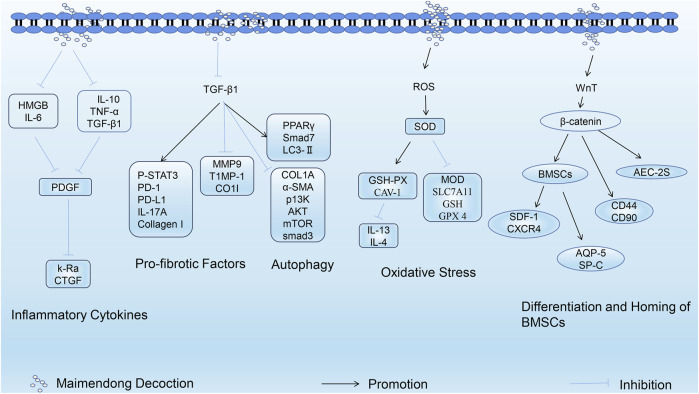
Mechanism of Maimendong Dection in the treatment of pulmonary fibrosis (the mechanism of MMDD in the treatment of Pulmonary fibrosis is mainly focused on reducing the level of inflammatory cytokines, inhibiting the expression of pro-fibrotic factors, inhibiting oxidative stress injury, promoting the differentiation and homing of bone marrow mesenchymal stem cells, and enhancing cell autophagy activity).

In summary, MMDD exerts anti-PF effects by reducing levels of inflammatory cytokines, inhibiting the expression of profibrotic factors, inhibiting oxidative stress damage, promoting the differentiation and homing of BMSCs, and enhancing autophagy activity (Figure 2). However, there are only a few studies related to the treatment of PF using MMDD, primarily focusing on *in vivo* experiments. In the future, more emphasis will be placed on cell experiments to explore additional mechanisms of action of MMDD in the treatment of PF.

## 5 Pharmacological study of MMDD in PF treatment

### 5.1 Ophiopogonis

*Ophiopogonis* is the dried root of *liliaceae Ophiopogon japonicus*. It has a sweet and slightly bitter taste, as well as a slightly cold nature. It is commonly used to treat cough (Chen et al., 2016), tumor (Liu et al., 2023), diabetes and its complications (Mao et al., 2020), cardiovascular and cerebrovascular diseases (Yang et al., 2020), and other ailments. Modern pharmacological studies have discovered various beneficial effects of *ophiopogonis*, including anti-heart failure and anti-tumor properties, regulation of gastrointestinal smooth muscle and intestinal flora, protection of the nervous system, improvement of liver and lung injuries, anti-inflammatory and antioxidant effects, hypoglycemic and anti-aging properties, as well as immune regulation (Shen et al., 2020). The main chemical components of *ophiopogonis* include steroidal saponins, polysaccharides, amino acids, and high isoflavone compounds (Wang et al., 2021; Zha et al., 2022; Lei et al., 2024). Additionally, *ophiopogonis* and its extracts have demonstrated protective effects against lung injury (Wang et al., 2020). Studies have shown that ophiopogonin D, an active ingredient in *ophiopogonis*, plays a role in treating PF by promoting PINK1/Parkin-dependent mitophagy in lung tissue and improving mitochondrial function (Peng et al., 2023). Furthermore, ophiopogonin C can reduce collagen deposition and the accumulation of inflammatory cells and fibroblasts in mice with PF, decrease the levels of pro-fibrotic cytokine TGF-β1, and increase SOD activity in lung tissue. These effects ultimately alleviate mortality and fibrosis levels in mice (Fu et al., 2022).

### 5.2 Pinellia

*Pinellia* is the dried tuber of *Pinellia ternata*, a plant belonging to the Araceae family. It possesses spicy and warm properties, but it is also toxic. *Pinellia* is often used in the treatment of peptic ulcers (Zhu et al., 2021), sleep disorders (Lin et al., 2019), hypertension, and other diseases (Jiang et al., 2021). Modern pharmacological studies have revealed that it has anti-inflammatory, antitumor, sedative, hypnotic, antitussive, and expectorant effects; the main chemical components include alkaloids, organic acids, amino acids, flavonoids, volatile oils, sterols, polysaccharides, and other compounds (Ting et al., 2023). Studies have demonstrated that raw *pinellia* can alleviate inflammatory infiltration, restore alveolar structure, reduce collagen deposition, and decrease hydroxyproline content in lung tissue of mice with PF(He et al., 2022). These effects may be related to peptides and organic acids present in *pinellia* decoction, which provides direction for further investigation into the pharmacodynamic molecules of *pinellia* (Xue et al., 2021). Flavonoids baicalin and baicalein, found in *pinellia*, exhibit therapeutic effects on PF(Ting et al., 2023). Baicalein has been shown to inhibit TGF-β1-induced lung fibroblast differentiation by downregulating miR-2 and connective tissue growth factor (CTGF), thereby reducing the production of type I collagen (ColI) in lung tissue (Cui et al., 2018; Sun et al., 2020). On the other hand, baicalin inhibits the TGF-β1-induced extracellular signal-regulated kinase 1/2 (ERK1/2) signaling pathway, lung fibroblast and fibroblast proliferation, and reduces Ca^2+^ concentration, thus achieving an anti-PF effect (Huang et al., 2016; Zhao et al., 2020).

### 5.3 Ginseng

*Ginseng* refers to the dried root and rhizome of the Araliaceae plant ginseng. It possesses a sweet and slightly bitter taste and has a slightly warm nature. It is commonly used in the treatment of diabetes (Juan et al., 2023), cardiovascular disease (Lanchun et al., 2022), breast cancer (Hamidian et al., 2023). Pharmacological studies have identified several beneficial effects of *ginseng*, including antioxidant, anti-inflammatory, antibacterial, anti-cardiovascular, and anti-diabetic properties. The main active components of g*inseng* are ginsenosides, volatile oil, and polysaccharides (Ratan et al., 2021). Ginsenosides, in particular, play a key role in its therapeutic effects against PF. They can downregulate the expression of TGF-β1, Smad2, Smad3, MMP-2, MMP-9, and metalloproteinase-1 inhibitors, while upregulating the expression of Smad7 protein. These effects contribute to the anti-PF effects of *ginseng* (Yang et al., 2019). Ginsenoside Rg3, a steroidal saponin found in *ginseng*, can improve lung function by reducing collagen deposition and the expression of mesenchymal markers in lung tissue (Lee et al., 2020). Deng et al. (2023) found that ginsenoside Rg1 has been shown to slow down the progression of PF by inhibiting the expression of cysteine aspartate protease-1, thus inhibiting cell pyroptosis, a novel inflammatory cell death mode. Additionally, ginsenoside AD-1 exhibits anti-PF effects by improving apoptosis and inhibiting the expression of TGF-β1, α-SMA, Sirtuin 3 (Sirt3), and other proteins (Su et al., 2021).

### 5.4 Glycyrrhiza

*Glycyrrhiza* refers to the dried root and rhizome of the sweet and flat legume glycyrrhiza plant. It is commonly used in the treatment of lung cancer (Rasha et al., 2022), ulcerative colitis (Lu et al., 2022), depression (Cao et al., 2020) and other diseases. *glycyrrhiza* contains various chemical constituents, including flavonoids, saponins, alkaloids, amino acids, coumarins, and polysaccharides, these extracted and isolated compounds have demonstrated diverse pharmacological effects, such as anti-tumor, anti-viral, anti-bacterial, anti-inflammatory, and immune regulatory activities (Yang et al., 2017; Dheeraj et al., 2022). Research has shown that *glycyrrhiza* and some of its extracts possess anti-PF effects (Ghorashi et al., 2016). One of its saponin components, glycyrrhizic acid, has been found to inhibit the increase of TGF-β1, IL-17, and p-Smad2 expression in mice with PF, thus exerting anti-fibrotic effects by suppressing the expression of transforming growth factor and inflammatory factors (Li et al., 2018; James et al., 2022). Another compound, licochalcone A, a phenolic chalcone derived from *glycyrrhiza* significantly inhibits TGF-β-induced transformation of fibroblast MRC-5 cells and reduces the expression of α-α-SMA and fibronectin (FN) in lung tissue. This indicates that Licochalcone A achieves anti-PF effects by blocking the TGF-β/Smad signaling pathway and inhibiting fibroblast transformation (Fu et al., 2019). Isoliquiritigenin, a flavonoid component of *glycyrrhiza* extract (Miyamura et al., 2021), has been observed to significantly inhibit the proliferation, migration, and morphological changes of A549 cells induced by TGF-β1. It increases E-cadherin levels while reducing the expression of N-cadherin, Vimentin, α-SMA, FN, EMT-related transcription factors, and phosphorylated Erk1/2. These effects help maintain cell epithelioid morphology and reduce the production of lung fibroblasts (Cai et al., 2022).

### 5.5 Jujuba

*Jujuba*, the dry and mature fruit of the Rhamnaceae plant jujube, is widely used in clinical practice for treating various diseases such as cardiovascular diseases (Zahra et al., 2019), liver cancer (Guo et al., 2016), and rectal cancer (Ruan et al., 2023). It possesses a sweet and warm nature, and its chemical components consist of triterpenoids, saponins, flavonoids, alkaloids, glycosides, nucleosides, vitamins, steroids, and other compounds (Liu et al., 2015). *Jujuba* exhibits pharmacological effects including antibacterial, antioxidant, sedative, hepatoprotective, antihyperglycemic, and antihyperlipidemic activities (Zahra et al., 2019). Flavonoids are one of the key chemical components found in jujube (Ren et al., 2022), and quercetin belongs to flavonoids (Baby et al., 2021). Studies have demonstrated that fibroblast senescence contributes to the pathogenesis of PF. Quercetin has been found to reverse the resistance to death ligand-induced apoptosis by promoting the expression of FasL receptor and caveolin-1, inhibiting AKT activation, restoring the sensitivity of senescent fibroblasts to pro-apoptotic stimulation, and reducing bleomycin-induced PF in elderly individuals (Hohmann et al., 2019). Moreover, quercetin can improve PF by inhibiting the SphK1/S1P signal transduction (Zhang et al., 2018). Another compound present in *jujuba*, ursolic acid, which is a terpenoid, also exhibits an anti-PF effect. Ursolic acid inhibits CASP3 to alleviate inflammation, thereby slowing down the occurrence of PF(Ma et al., 2019). Chen and Tian (2019) discovered that ursolic acid significantly reduces macrophage infiltration and lymphocyte exudation in the alveolar cavity and alveolar septum, thereby reducing PF. This effect may be attributed to the inhibition of the Toll-like receptor 4 (TLR4)/nuclear factor-kappaB (NF-κb) signaling pathway and the subsequent release of inflammatory factors.

The study found that MMDD contains flavonoids, saponins, alkaloids, terpenoids, phenylpropanoids and other compounds, among which flavonoids mainly come from *ophiopogonis*, *glycyrrhiza*, and *jujuba*; Terpenoids are mainly derived from *ginseng, glycyrrhiza*, *jujuba*, and *oryza sativa*; steroid compounds are mainly derived from *ophiopogonis*; phenylpropanoid compounds are mainly from *pinellia*, *oryza sativa*, *glycyrrhiza* and *jujuba* (Yang et al., 2020). The active ingredients in MMDD for treating PF encompass flavonoids, saponins, terpenoids, and alkaloids (Ren et al., 2022). These include ophiopogonin C, ophiopogonin D, baicalin, baicalein, ginsenoside Rg3, ginsenoside Rg1, glycyrrhizic acid, licochalcone A, isoliquiritigenin, ursolic acid, oleanolic acid, and quercetin (Table 2), primarily sourced from *ophiopogonis, pinellia, ginseng, glycyrrhiza* and *jujuba*. Currently, there has been no relevant research on using *oryza sativa* for PF treatment. Therefore, further investigation is required to determine whether the chemical components and extracts of *oryza sativa* possess therapeutic effects for PF.

**TABLE 2:** Effective chemical constituents of Maimendong Decoction in the treatment of pulmonary fibrosis.

NO	Compound	Formula	Molecular weight	Chemical structure	References
1	Ophiopojaponin D	C_44_H_70_O_16_	855.017	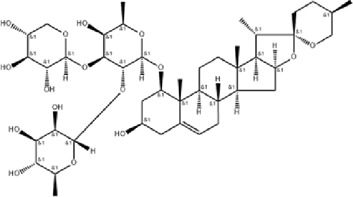	Lei et al. (2024)
2	Ophiopogonin C	C_44_H_70_O_18_	887.016	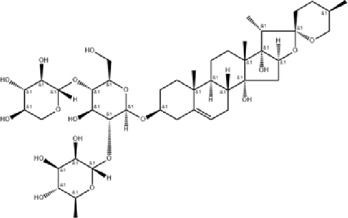	Zha et al. (2022)
3	Baicalin	C_15_H_10_O_5_	270.24	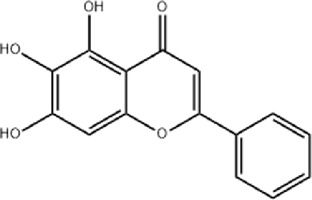	Xue et al. (2021), He et al. (2022)
4	Baicalein	C_21_H_18_O_11_	446.37	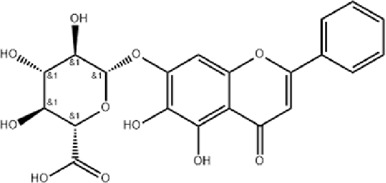	Cui et al. (2018), Sun et al. (2020)
5	Ginsenoside Rg3	C_42_H_72_O_13_	785.03	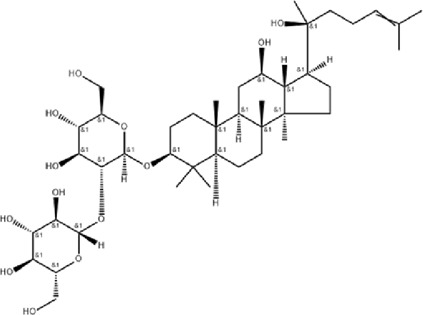	Ratan et al. (2021)
6	Ginsenoside Rg1	C_42_H_72_O_14_	801.013	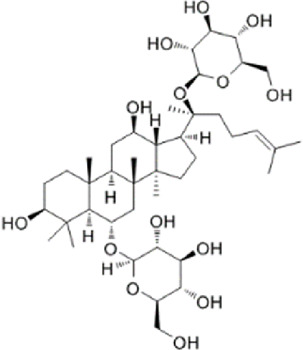	Yang et al. (2019)
7	Glycyrrhizic acid	C_42_H_62_O_16_	822.94	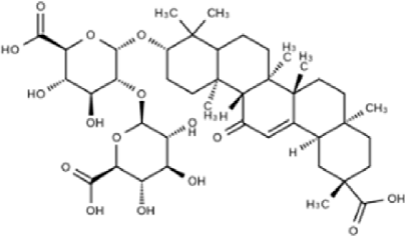	Ghorashi et al. (2016), Dheeraj et al. (2022)
8	Licochalcone A	C_21_H_22_O_4_	338.4	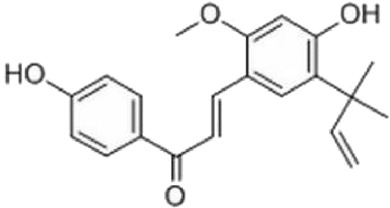	Li et al. (2018)
9	Isoliquiritigenin	C_15_H_12_O_4_	256.25	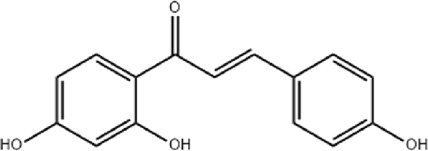	Fu et al. (2019)
10	Quercetin	C_15_H_10_O_7_	302.236	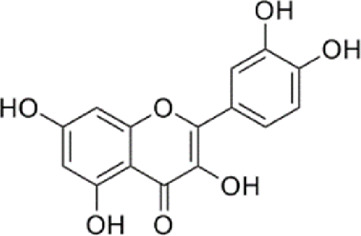	Hohmann et al. (2019), Baby et al. (2021)
11	Ursolic Acid	C_30_H_48_O_3_	456.71	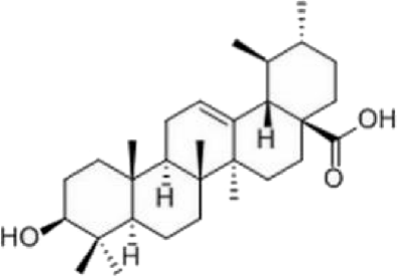	Zhang et al. (2018), Ma et al. (2019)

## 6 Summary and outlook

The treatment of PF in TCM is primarily focused on addressing deficiency, phlegm, and blood stasis. A representative prescription for PF treatment is MMDD, where each medicine in the prescription plays a complementary role. MMDD has shown significant effectiveness in treating PF, with its mechanism confirmed to involve reducing inflammatory cytokine levels, inhibiting pro-fibrotic factor expression, suppressing oxidative stress injury, promoting differentiation and homing of BM-MSCs, and enhancing autophagy activity. While many scholars have confirmed the efficacy of MMDD in alleviating PF, there are still some areas that require improvement. Firstly, although significant therapeutic effects have been observed *in vitro* and animal experiments, clinical studies are limited. Therefore, future research should focus on increasing clinical investigations. Secondly, the animal models used for PF induction need refinement. Currently, bleomycin and TR-induced models are commonly used, but there are variations in treatment efficacy among different drug-induced models, which may not accurately reflect the symptoms of clinical PF patients. This discrepancy is a common limitation in disease modeling. Thirdly, current research primarily focuses on understanding the mechanisms of PF without thoroughly exploring the corresponding targets of the disease. Further research can delve into studying the potential targets of MMDD’s components for treating PF. Fourth, there has been currently no research on using *oryza sativa* for treating PF, and it remains unknown whether *oryza sativa* and its extracts contain important components for PF treatment. Identifying more chemical components of MMDD in treating PF will be crucial for informing clinical approaches. Fifth, In recent years, with the wide application of traditional Chinese medicine, traditional Chinese medicine is shoddy, and the problem of false chaos begins to appear, and the quality of traditional Chinese medicine is uneven, which leads to the failure of the patient to achieve the expected effect after taking the medicine, and may produce certain side effects, so in order to achieve good efficacy, relevant departments should improve the quality evaluation system of traditional Chinese medicine, formulate relevant evaluation standards, and reduce the occurrence of safety problems of traditional Chinese medicine. Lastly, Most clinical studies have shown that MMDD has a significant effect in the treatment of pulmonary fibrosis, but it does not mention its adverse reactions, because some traditional Chinese medicine will lead to liver injury in the treatment of diseases, such as Pinellia has hepatotoxicity, and the material basis and mechanism of hepatotoxicity are not yet clear, so the clinical use of MMDD should not only consider the severity of the patient‘s condition, but also consider the hepatotoxicity of Pinellia. It is necessary to select the appropriate dose of Pinellia according to the severity of the patient‘s condition to avoid liver injury.

Although the single herb in MMDD contains anti-pulmonary fibrosis chemicals, there is no research on the main active ingredients of MMDD in the treatment of PF. Some scholars speculate that β-sitosterol, stigmasterol, kaempferol, fumarine, mairin, quercetin, and ginsenosides may be the components of MMDD to inhibit PF, but there is no relevant mass spectrometry detection, so metabolomic analysis of MMDD can be carried out in the future to find the main active ingredients of MMDD in the treatment of PF. Due to the complex mechanism of PF and the characteristics of multi-component and multi-target of traditional Chinese medicine, biotechnology such as network pharmacology and metabolomics can be used to clarify the active ingredients and corresponding disease targets of MMDD in the treatment of PF in the future, so as to provide a reference for the development and clinical treatment of traditional Chinese medicine.
